# The fecal mycobiome in patients with Irritable Bowel Syndrome

**DOI:** 10.1038/s41598-020-79478-6

**Published:** 2021-01-08

**Authors:** A. Das, E. O’Herlihy, F. Shanahan, P. W. O’Toole, I. B. Jeffery

**Affiliations:** 1 4D Pharma Cork Limited, Cavanagh Pharmacy Building, University College Cork, National University of Ireland, Cork, Ireland; 2 School of Microbiology, University College Cork, National University of Ireland, Cork, Ireland; 3 APC Microbiome Ireland, University College Cork, National University of Ireland, Cork, Ireland

**Keywords:** Fungi, Microbial communities, Microbiome, Irritable bowel syndrome

## Abstract

Alterations of the gut microbiota have been reported in various gastrointestinal disorders, but knowledge of the mycobiome is limited. We investigated the gut mycobiome of 80 patients with Irritable Bowel Syndrome (IBS) in comparison with 64 control subjects. The fungal-specific internal transcribed spacer 1 (ITS-1) amplicon was sequenced, and mycobiome zero-radius operational taxonomic units (zOTUs) were defined representing known and unknown species and strains. The fungal community was sparse and individual-specific in all (both IBS and control) subjects. Although beta-diversity differed significantly between IBS and controls, no difference was found among clinical subtypes of IBS or in comparison with the mycobiome of subjects with bile acid malabsorption (BAM), a condition which may overlap with IBS with diarrhoea. The mycobiome alterations co-varied significantly with the bacteriome and metabolome but were not linked with dietary habits. As a putative biomarker of IBS, the predictive power of the fecal mycobiome in machine learning models was significantly better than random but insufficient for clinical diagnosis. The mycobiome presents limited therapeutic and diagnostic potential for IBS, despite co-variation with bacterial components which do offer such potential.

## Introduction

The gut microbiota has been implicated in various gastrointestinal and extra-intestinal diseases, including Irritable Bowel Syndrome (IBS)^1–6^. Most studies have focused on the gut microbes (Archaea, and eubacteria), but little is known about the role of eukaryotes (mainly fungi) in health and disease.

Because of its clinical heterogeneity and the unclear aetiology of IBS, robust biomarkers and therapeutic targets for IBS are difficult to identify^7^. Multiple possible causes or triggers including bacterial dysbiosis, altered activity of the gut-brain axis, and environmental factors like food, may contribute to the pathophysiology of IBS^5,8,9^. One such factor which is understudied, and which has recently attracted attention, is the gut mycobiome (the fungal community of the gut). In recent studies, an altered gut mycobiota in IBS has been reported^7,10–15^, but this has been based on small study populations and disparate analytical methods.

Furthermore, targeting the mycobiome for treatment of IBS specific symptoms bears certain caveats. For example, Candida-associated infectious diarrhoea due to overgrowth of Candida also presents IBS-like symptoms^16^, and some studies showed absence of any conclusive link between Candida overgrowth and IBS^17^. Also, Candida abundance is directly associated with short term high carbohydrate consumption^7^. Such mycosis may lead to a false diagnosis of IBS, and its incorrect treatment^18^. Symptoms like visceral hypersensitivity are observed only in a subset of subjects with IBS and this has been associated with the gut mycobiome^10^. Therefore, treatment with antifungal agents to ameliorate such symptoms may benefit only a subset of IBS sufferers and may not universally cure IBS. Thus, it is imperative to thoroughly investigate and establish the gut mycobiome as an potential target for IBS therapy, since there is very little evidence of role of gut mycobiome in IBS^16^.

We analysed the fecal mycobiome composition by sequencing the ITS1 region amplified from the fecal DNA of patients with IBS and from control subjects. We also assessed the association of the mycobiome with our previously published bacteriome and metabolome datasets^6^. The results suggest that despite a significant difference in the mycobiome between IBS and controls, it is a weak predictive biomarker for IBS.

## Results

### The fecal mycobiome in IBS and controls

Next generation sequencing of the fungal ITS-1 region amplicon was performed on 144 (IBS; n = 80 and control: n = 64) fecal samples. The sequence data returned yielded 10,498,738 (median: 68,044, IQR: 57,684–87,378) read pairs of length 251 base pairs (bp). After data pre-processing, 70.2% or 7,368,688 (median: 46,099, IQR: 39,274–59,185) read pairs were retained and had a median length of 254 bp (IQR: 188–442). Of these reads, 95.2% or 7,013,893 (median: 44,631, IQR: 37,051–56,672) mapped to 1631 fungal zOTUs (per sample median: 60, IQR: 46–75). Across the IBS and control samples, the genera *Saccharomyces*, *Candida*, *Debaryomyces*, and *Penicillium* were the most abundant fungal genera. The variation in taxonomic prevalence was found to be sample specific (Fig S1).

**Figure 1:**
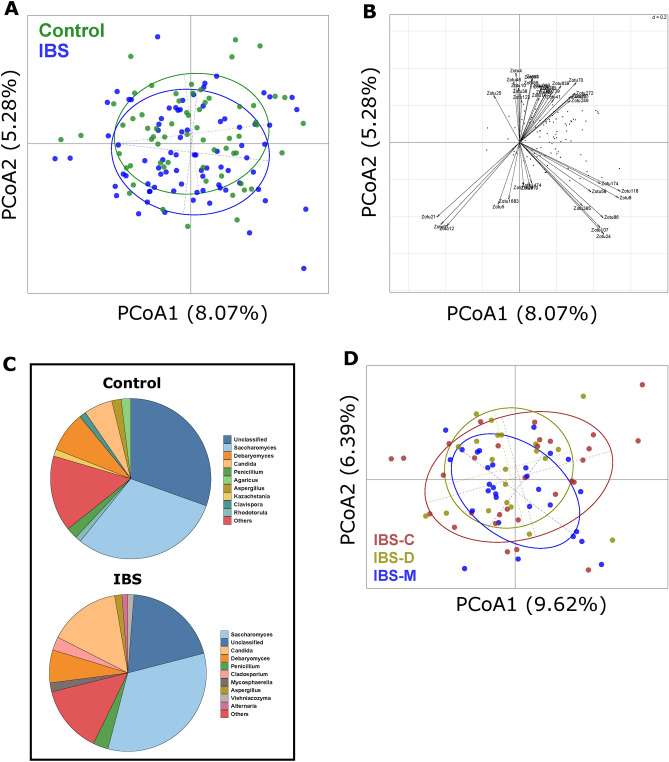
Mycobiome compositional differences between IBS and Control subjects. (**A**) Principal coordinate analysis of sample data (n = 144) using Bray–Curtis distance using log10 transformed abundance of zOTUs. A significant separation was observed between IBS (n = 80), and control (n = 64) samples, along the second principal axis. Significance was determined using permutational multivariate analysis of variance based on Bray–Curtis distance (Adonis; p-value = 0.004). (**B**) Features and their correlation with second principal axis, showing direction of association. Features showing significant spearman correlation (adj p < 0.05) between their abundance and second axis are shown with labelled arrows. (**C**) Pie charts showing top 10 abundant fungal genera in Control (n = 64) and IBS populations (n = 80). (**D**) Principal coordinate analysis based on Bray Curtis distance and log10 transformed zOTU abundance profile of clinical sub-types of IBS population. No significant differences were observed between the 3 clinical subtypes (IBS-C: n = 30, IBS-D: n = 21 and IBS-M: n = 29), (Adonis; p-value = 0.694).

Beta-diversity of IBS and controls was investigated using Principal Coordinate Analysis (PCoA) (Fig. 1A). Permutational MANOVA showed significant differences between the mycobiome of IBS and controls (p-value = 0.004), with IBS and the control samples separating along the secondary principal axis. To identify features associated with this split, the abundances of the fungi were correlated with the second axis. Features identified as significant are presented in Fig. 1B. Among the zOTUs significantly and positively correlated with the second principal axis, seven out of sixteen zOTUs belonged to the genera *Candida*, and *Saccharomyces*. Of these, two zOTUs (one classified as *Candida* spp. and another as *Candida albicans*), were significantly more abundant in IBS (p-value < 0.05). Three zOTUs classified as *Hanseniaspora ovarum*, commonly found in fresh fruits^19^, were significantly less abundant in IBS (p-value < 0.05). A total of thirteen out of twenty-three fungal zOTUs that were significantly negatively associated with the IBS-control separation belonged to (at the genus level) *Penicillium*, *Agaricus*, and *Hanseniaspora*.

Genus level differences in abundances of the mycobiome were detected between the IBS and control subjects (Fig. 1C). Statistical analysis of IBS versus controls identified that *Malassezia*, *Candida*, and *Cladosporium* at the genus level were significantly more abundant in IBS (p-value < 0.05). No significant difference was observed between the IBS clinical subtypes (p-value = 0.694; Fig. 1D). These differences must be placed in the context of the high level of variance in mycobiome feature abundance seen within the IBS and control groups.

**Figure 2:**
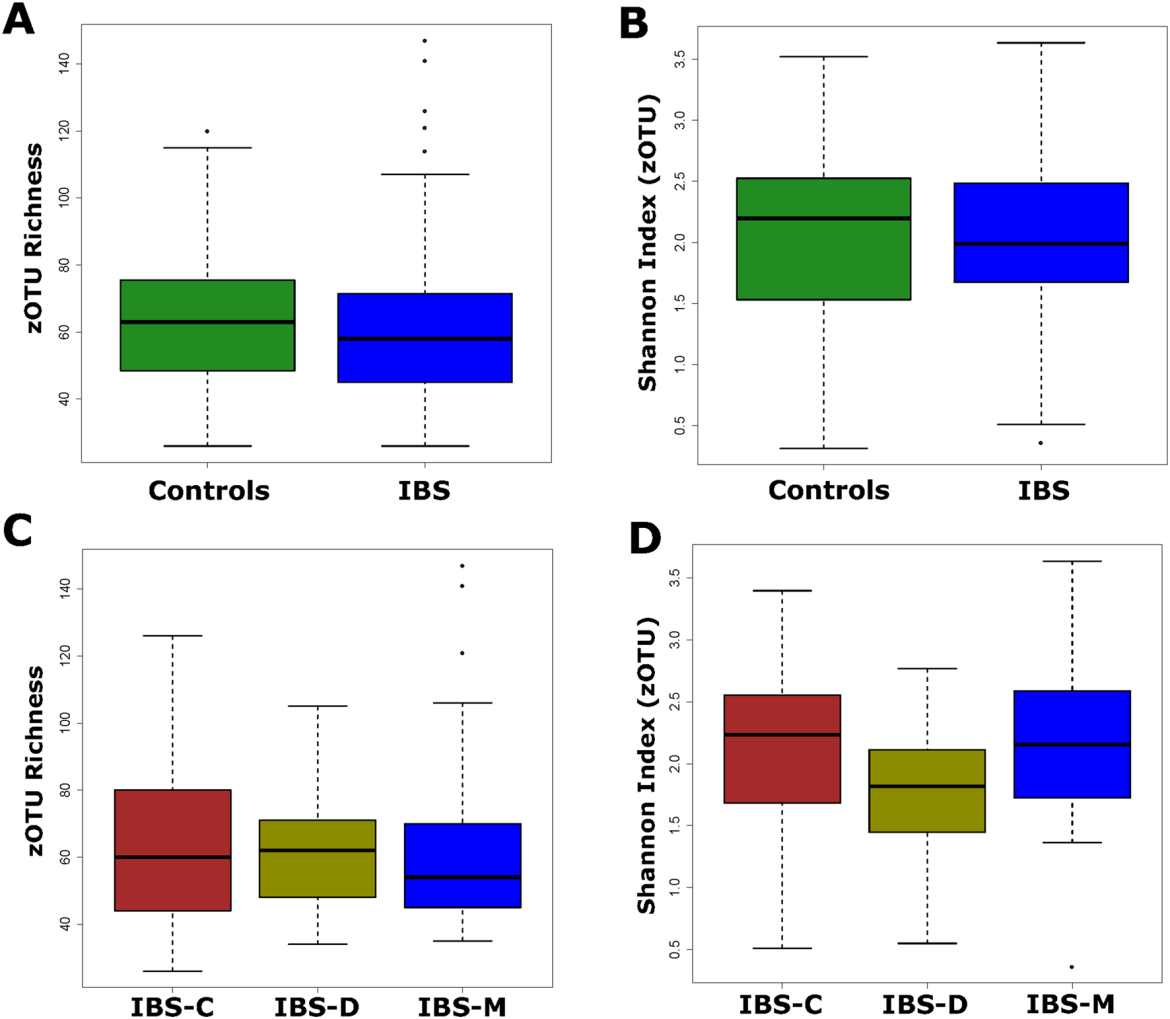
Mycobiome diversity differences in the analysed population. Two diversity measures were calculated, viz., Richness, and Shannon diversity index. None of the differences were significant. (**A**) and (**B**) zOTU richness, and Shannon diversity differences between IBS (n = 80), and control (n = 64) populations. Wilcoxon rank sum test returned nonsignificant p-values of 0.301 and 0.84, respectively. (**C**) and (**D**) zOTUotu richness, and Shannon diversity differences between clinical subtypes of IBS (IBS-C: n = 30, IBS-D: n = 21, IBS-M: n = 29). Kruskal–Wallis test returned non-significant p-values of 0.75 and 0.17, respectively.

Diversity analyses showed no significant differences in richness or Shannon diversity between IBS and controls (Fig. 2A and B), or among the IBS clinical subtypes (Fig. 2C and D). Independent of clinical stratification, it is interesting to note that the largest trend as defined by the first principal component was based on differences in alpha-diversity (Fig S2 A and B). Therefore, mycobiome diversity rather than taxonomy explains a major part of the variance in the dataset. While both Shannon diversity and richness showed significant positive correlation with the first axis, and the coefficient was much larger for the latter (Spearman rho: 0.596, 0.794 respectively).

### Predictive value of the mycobiome for IBS

**Figure 3:**
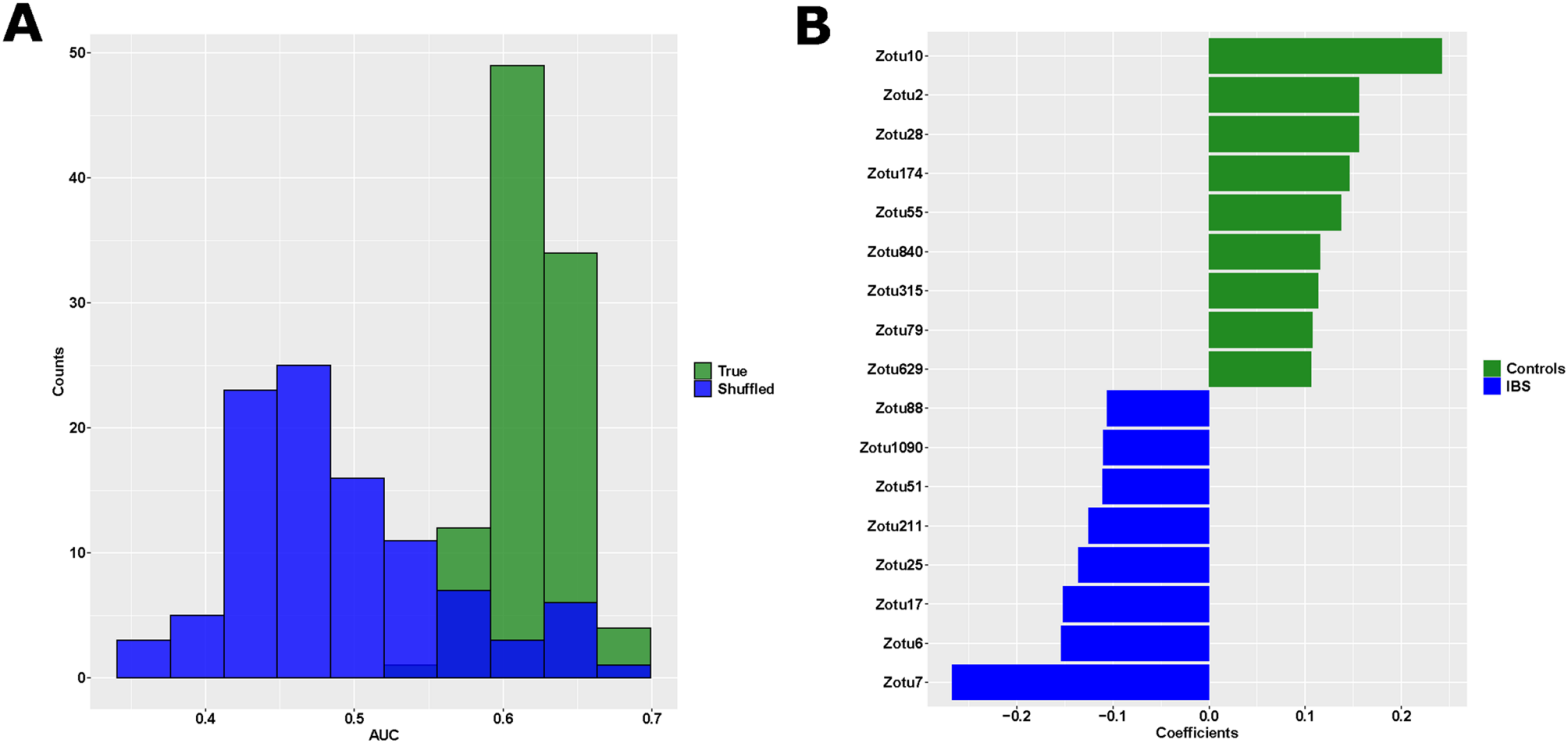
Predictive power of mycobiome to distinguish between IBS and controls. (**A**) Distribution of mean Area under the curve (AUC) of each tenfold cross validation, across 100 iterations, using true, and shuffled classes. The difference was significant between True (0.619 ± 0.025), and Shuffled (0.489 ± 0.071) classes (p-value < 0.001). B) Feature coefficients from trained models, significantly different from random are shown. zOTUs with negative coefficients are associated with IBS, and those with positive coefficients are associated with controls (green).

We investigated the power of the mycobiome to predict IBS using a supervised machine learning model, Support Vector Machine (SVM) with a tenfold cross validation. The returned values for sensitivity, specificity and area under the curve (AUCs) varied depending on the random seed used and therefore we repeated the analysis for 100 iterations where the class labels were maintained and compared to 100 iterations where the class labels were randomised. Based on Receiver Operating characteristics (ROC) curve analysis, the obtained AUC using true class labels was significantly different from shuffled labels (Fig. 3A), suggesting that the prediction results obtained were not by chance. However, although significantly better than random, the classifier performance was not robust enough to distinguish between IBS and controls to diagnostic level accuracies. While the AUC (0.619 ± 0.025), and specificity (0.355 ± 0.053) were significantly higher than random, the sensitivity (0.776 ± 0.032) was significantly lower than random. SVM coefficients, univariate statistics, and other information including taxonomic classification are summarized in Table S1.

Feature coefficients from corresponding models (True and shuffled classes) were obtained and compared using the z-test using package BSDA in R. Feature coefficients that were significantly different from random (adjusted p-value < 0.05, Benjamini–Hochberg FDR) are shown in Fig. 3B. The two most predictive mycobiome zOTUs identified were zOTU7 (classified as *Cladosporium cladosporioides*) which was more abundant in patients with IBS, and zOTU10 (an unidentified fungus) which was less abundant in IBS. These two zOTUs were confirmed to be significantly differentially abundant based on Wilcoxon rank sum test (Fig S3A and B).

### Co-variance of the mycobiome with fecal metabolome and bacterial metagenome

**Figure 4:**
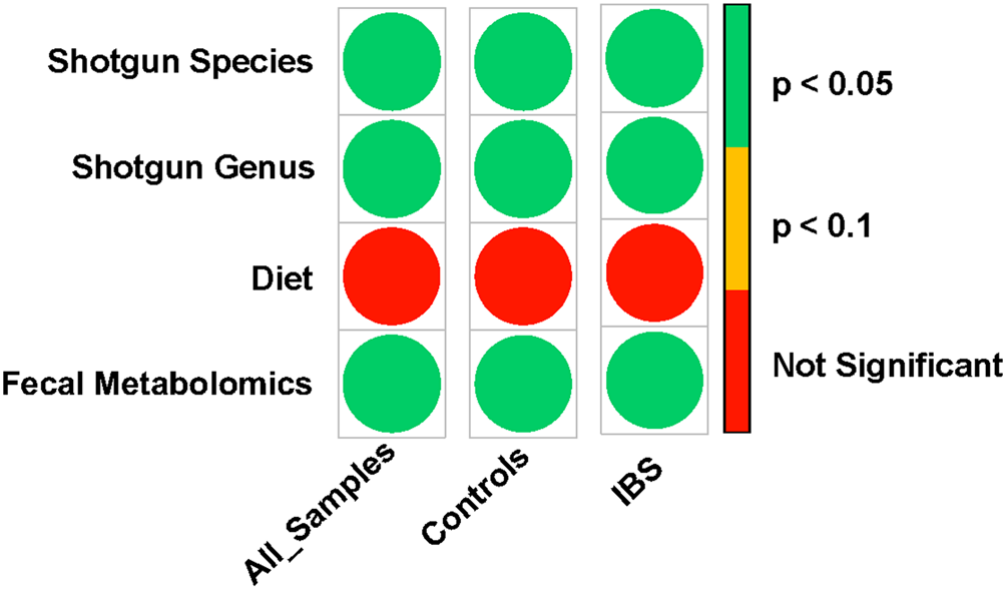
Co-inertia analysis between mycobiome composition with bacterial composition (All subjects: n = 138, controls: n = 58, IBS: n = 80), food consumption (All subjects; n = 144, controls: n = 64, IBS: n = 80), and fecal metabolome (All subjects; n = 142, controls: n = 62, IBS: n = 80). Mycobiome showed significant association with bacterial composition and fecal metabolome but failed to show significance with food consumption. Degree of association as RV coefficient is presented in supplementary Table S2.

Co-inertia analysis based on PCA was performed to examine concordance between the fecal mycobiome and bacteriome, metabolome, and habitual dietary intake across the cohort and within IBS and control populations (Fig. 4, Table S2). Significance was tested using monte-carlo permutation methodology. The mycobiome showed significant co-variance with the bacteriome, and metabolome, but not with diet. Co-variance between the mycobiome and metabolomic and metagenomic data was higher in control subjects than in IBS patients (Table S2).

To identify the variables that co-vary between the bacterial metagenome and the mycobiome, the co-inertia loadings of the two datasets were visualized (Fig. S4). The ordination of mycobiome and bacterial metagenomic datasets based on co-inertia are shown in Figure S4A and B, respectively. Features in the direction of positive y-axis are associated with controls, while features in the opposite direction are associated with IBS (Fig S4C and D). Mycobiome zOTUs (Fig S4C) lying in the same direction as shotgun species (Fig S4D) co-vary. Similarly, mycobiome and the fecal metabolome samples were visualized, based on co-inertia (Fig S5A, and B). Mycobiome zOTUs (Fig S5C), and fecal metabolites (Fig S5D) in the direction of negative x-axis are associated with controls, while features lying in the opposite direction are associated with IBS. Further details of the feature IDs and names of the metabolites are given in Table S3 and the taxonomy of the zOTUs are listed in Table S1. These putative associations may indicate the major drivers of stratification within the datasets and the concordance between them, but more functional investigation is required to confidently infer the biological importance of these associations.

**Figure 5:**
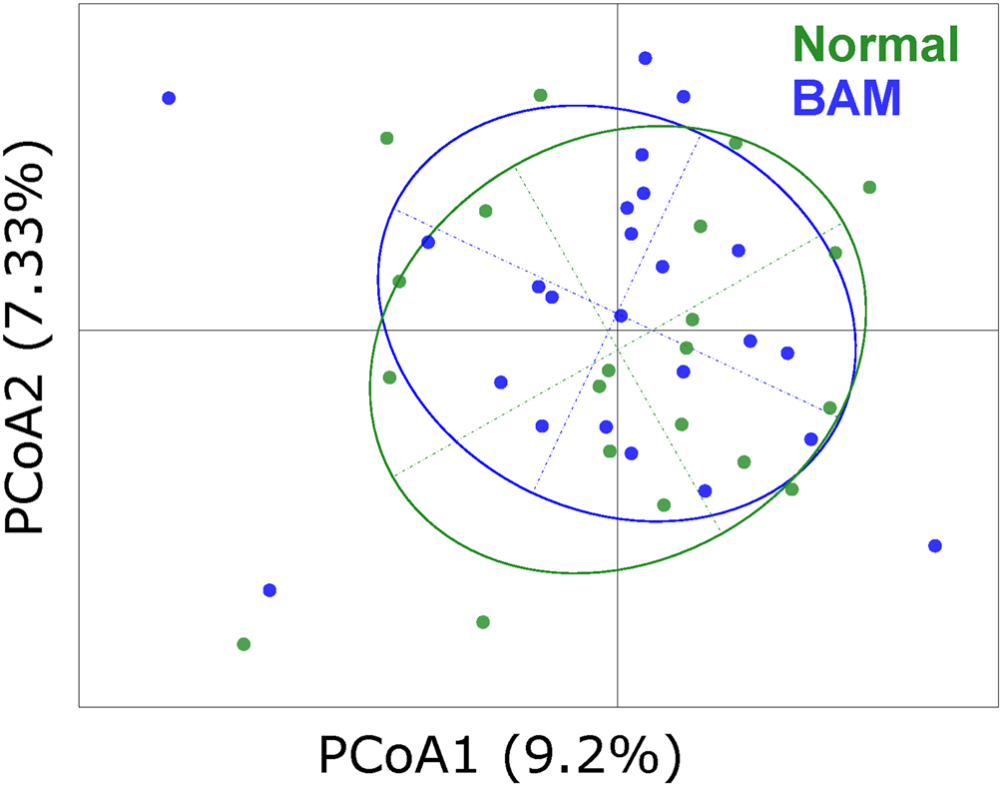
Principal coordinate analysis of IBS patients who had undergone SeHCAT assay to detect bile acid malabsorption (BAM). Samples were categorized as normal (n = 21) (> 20% SeHCAT retention) or having borderline to severe BAM (< 20% SeHCAT retention) (n = 24). There was no significant difference between the BAM classes (Adonis p-value = 0.985).

Bile acid malabsorption (BAM) is a condition which causes diarrhoea and which is difficult to distinguish from patients with diarrhoea-predominant IBS^20^. In our previous analysis, we observed that only the bacteriome of subjects with severe BAM (SeHCAT retention < 5%), showed a significant separation from that of patients in other BAM severity categories^6^. To investigate the differences in mycobiome between BAM classes, a subset of IBS patients (n = 45) who underwent a SeHCAT assay to detect BAM (reported previously by Jeffery et al.^6^), were analysed. No significant difference in the overall mycobiome was observed between BAM positive (≤ 20% SeHCAT retention) and normal bile acid retention groups (Fig. 5).

## Discussion

This study shows that the fecal mycobiome varies considerably from person to person both in alpha-diversity and in its taxonomic composition. Nevertheless, the mycobiome differentiates patients with IBS from a control population. There were no differences in the mycobiome among the clinical subtypes of IBS or in the patients with BAM. We observed significant co-variation between mycobiome, and bacterial metagenome and fecal metabolome.

Intolerance to food and triggering or exacerbation of IBS symptoms is known to occur in some patients^21^. Such symptoms, including abdominal distress, are attributed to the intolerance of IBS patients to diet that is high in FODMAP content^21^. Therefore, a low FODMAP diet is sometimes recommended to patients with IBS. In our previously published study, we observed that clusters of food items high in FODMAP content were associated with the Control group^6^. Identification of depleted levels in IBS subjects of a yeast (*Hanseniospora ovarum*) which is commonly found in fresh fruits, appears to be in accordance to the food correlation data. However, there was no association with the overall dietary data, therefore suggesting no link between food intake and the fungal component of the microbiota. This contrasts with some published reports which linked habitual diet to mycobiome composition^22,23^.

Multi-omic integrative studies pertaining to IBS are limited, but some reports show applicability of integrating bacteriome and metabolome for a better understanding of the disorder as potential biomarkers or a means to stratify patients^6,24^. Our analysis suggests that the mycobiome is a poor diagnostic biomarker for IBS, but it could be a potential contributor to a diagnostic score if combined with the bacteriome and metabolome.

Due to the wide variance in the mycobiome taxonomic composition by abundance and by alpha diversity, we experienced a limited ability to identify specific taxa associated with the observed beta-diversity separation between IBS and controls. The current study population is from a single geographic region and further studies in larger populations and in other geographical regions are therefore required. In this initial investigation of the mycobiome, we sequenced the ITS-1 region, but future studies should consider sequencing both the ITS-1 and ITS-2 regions.

While the mycobiome may have pathogenetic importance in IBS, it does not seem to have a sufficiently distinct signature for diagnostic utility. However, it exhibits substantial heterogeneity and may identify clinically important disease subsets or help stratify patients if used in combination with the bacteriome and metabolome.

## Methods

### Study participants

Eighty patients with Irritable Bowel Syndrome (Rome IV Criteria), along with sixty-five control participants were recruited to this study. Both populations (IBS, and controls) belonged to same age range, ethnicity, and geographic region. The IBS population was further classified into clinical subtypes, viz., IBS-C (constipation predominant), IBS-D (diarrhoea predominant), and IBS-M (mixed). A subset of the IBS population (n = 45) who underwent a selenium-75 homocholic acid taurine (SeHCAT) assay for bile acid malabsorption detection were also analysed. A summary of the study population characteristics is given in Table 1. The study was approved by the Cork Research Ethics Committee, all research was performed in accordance with relevant guidelines/regulations, and all participants gave written informed consent to take part. Further details of participant recruitment, the metagenomic, fecal metabolomic, and dietary data used in this analysis is available in Jeffery et al.^6^.

**Table 1:** Characteristics of control and IBS study population.

	Control (n = 65)	IBS (n = 80)
Age range, years (mean)	19–65 (45)	17–66 (39)
Sex (male/female)	16/49	15/65
**BMI Class, n (%)**		
Normal	25 (38)	31 (39)
Obese Class I	11 (17)	14 (18)
Obese Class II	3 (5)	5 (6)
Obese Class III	1 (2)	3 (4)
Overweight	21 (33)	22 (28)
Underweight	3 (5)	3 (4)
**HADS: Anxiety, n (%)**		
Normal (0–10)	59 (91)	58 (73)
Abnormal (11–21)	6 (9)	22 (28)
**HADS: Depression, n (%)**		
Normal (0–10)	64 (98)	70 (88)
Abnormal (11–21)	1 (2)	10 (13)
**Bristol Stool Score, n (%)**		
Normal	54 (83)	18 (23)
Constipated	8 (12)	22 (28)
Diarrhoea	3 (5)	40 (50)
**IBS subtype, n (%)**		
IBS-C	N/A	30 (38)
IBS-D		21 (36)
IBS-M		29 (36)
SeHCAT assayed, n (%)	9 (14)	46 (58)
**Dietary group (FFQ), n (%)**		
Omnivore	63 (97)	74 (93)
Vegetarian	1 (2)	2 (3)
Pescatarian	1 (2)	1 (1)
Gluten-free	0 (0)	4 (5)
**Drinks alcohol, n (%)**		
Current	54 (83)	57 (71)
Previous	0 (0)	1 (1)
Never	10 (15)	22 (28)
**Smoker, n (%)**		
Current	10* (15)	14* (18)
Previous	13 (20)	18 (23)
Never	42 (65)	48 (60)

* 1 subject in each group smoked e-cigarettes.

BMI: Body Mass Index, FFQ: Food Frequency Questionnaire, HADS: Hospital Anxiety and Depression Scale, N/A: not applicable.

### Internal transcribed spacer (ITS) sequencing for mycobiome analysis

DNA was extracted from frozen fecal samples as described previously by Jeffery et al^6^ and prepared for ITS-1 sequencing for mycobiome analysis following the method by Huseyin et al.^25^. Briefly, DNA was extracted from 0.25 g of frozen fecal samples using the Qiagen DNeasy Blood and Tissue Kit according to manufacturer’s instructions. PCR was performed on 100 ng of DNA fecal extracts using Kapa Robust HotStart Readymix, ITS1 (Forward: 5′ -*TCGTCGGCAGCGTCAGATGTGTATAAGAGACAGCTTGGTCATTTAGAGGAAGTAA*—3′), and ITS2 (Reverse: 5′ – *GTCTCGTGGGCTCGGAGATGTGTATAAGAGACAGGCTGCGTTCTTCATCGATGC* – 3′) primers containing an Illumina adaptor sequence for indexing. Two technical replicates were pooled and then purified with AMPure SPRIselect. Forward and reverse barcodes were attached by a second round of adapter PCR using the 16S Sequencing Library Preparation Nextera protocol developed by Illumina (San Diego, California, USA). The resulting tagged samples were purified with AMPure SPRIselect, quantified using Qubit High Sensitivity dsDNA kit (Life Technologies) and equimolar amounts of each sample were then pooled before being sent for sequencing GATC Biotech AG, Konstanz, Germany on the MiSeq (2 × 250 bp) chemistry platforms.

### Fungal zOTU clustering and data processing

zOTUs (zero-radius Operational Taxonomic Units) were identified and profiles were generated using Usearch V11 pipeline codes. Firstly, raw reads were merged using default parameters, followed by primer sequence trimming and sequence orientation using in house python code and primer sequence information. Merged sequences, which were at least 100 base pairs long after primer trimming were retained and pooled into a single file for zOTU identification using UNOISE algorithm.

For zOTU identification, sequences were filtered based on base quality, overall sequence quality, and length, followed by dereplication to obtain unique sequences. These unique sequences were passed through the UNOISE algorithm, and zOTUs were obtained.

The pooled sequences prior to quality filtering were re-mapped to these zOTUs, and the abundance profile was generated.

An additional zOTU filtering step was performed owing to presence of zOTUs of bacterial origin. For this, bacterial genomes from Chocophlan database were pooled and sequence alignment of zOTUs were performed against it. zOTUs which mapped to bacterial genomes with high confidence and identity were rejected and a profile with 1631 zOTUs and their abundances in samples were obtained. The profile was normalized and filtered based on occurrence. zOTUs which were present in > 10% of samples were selected. The final profile with 138 zOTUs and 144 samples (IBS: n = 80, controls: n = 64) was transformed using logit function, or log10 transformation for subsequent analyses.

Logit transformation function:

$$f\left( x \right) = \ln \left( {\frac{x}{1 - x}} \right){\text{where, x is the normalized abundance of zOTU}}$$

Log10 transformation:

$$f\left( x \right) = log10\left( {x + 1E - 6} \right) + 6$$

where x is the scalar normalized abundance of zOTU and 1E − 6 is used as a minimal non-zero value and the addition of six ensure that all values are positive. Taxonomic classification of the representative sequences of zOTUs was done using UNITE database of fungal sequences, and qiime2′s feature-classifier, with default parameters.

### Statistical analysis

All statistical analyses were performed in R (v 3.5.1) with appropriate libraries mentioned. Beta-diversity was calculated using the Bray–Curtis dissimilarity metric and visualised with PCoA and co-inertia analysis. Significant differences in beta-diversity are defined by Permutational MANOVA as implemented by the Adonis functionality in the vegan R package. Spearman correlation was used to identify features that were significantly correlated with the PCoA secondary eigenvector. The p-values were adjusted using Benjamini–Hochberg FDR method, and adjusted p values < 0.05 were considered significant. The Wilcoxon rank sum test was used to identify significant differentially abundant features. Significance is defined as nominal unadjusted P-value of 0.05.

### Machine learning analysis

Machine learning classification was performed to investigate the predictive power of the mycobiome to distinguish between IBS and Controls, and to identify health-associated features. For this, SVMs with linear kernel were employed on the logit transformed abundance, using Caret package in R. Model training performed, were weighted based on class probabilities. tenfold cross validations for 100 iterations were performed, either using true labels, or shuffled class labels. For each tenfold CV using shuffled class labels, we obtained a distribution of coefficients for each feature, which was considered as null distribution. A single sample z test of this distribution was compared to the true coefficient values. Significantly different features after p value adjustments using Benjamini–Hochberg FDR method were identified and plotted (Fig. 3B).

### Co-inertia analysis

Association between the fecal mycobiome and other datasets (bacteriome, metabolome and diet) from the same study population previously reported by Jeffery et al.^13^ was performed using co-inertia analyses.

For association analysis of each dataset, samples common to the mycobiome and the compared dataset was used. For shotgun species/genus data from 138 study participants (IBS: n = 80, control: n = 58) was transformed using Hellinger transformation. The dietary data based on the Food Frequency Questionnaire (FFQ) from 144 individuals (IBS: n = 80, control: n = 64) was used in the analysis where the frequencies were converted to per week consumption of food items. For the fecal metabolome, profiles from 142 participants (IBS: n = 80, Control: n = 62) were included and these were log10 transformed. The mycobiome profiles in all the comparisons was log10 transformed.

## Supplementary Information

Supplementary Tables.

Supplementary Figures.
