# Supplementary material for: The fecal mycobiome in patients with Irritable Bowel Syndrome
Source: Sci Rep. 2021 Jan 8;11:124. doi: 10.1038/s41598-020-79478-6 (PMC7794320; doi:10.1038/s41598-020-79478-6)
Supplement: Supplementary file 2 — Supplementary Figures. [file 41598_2020_79478_MOESM2_ESM.pdf]

Figure S1

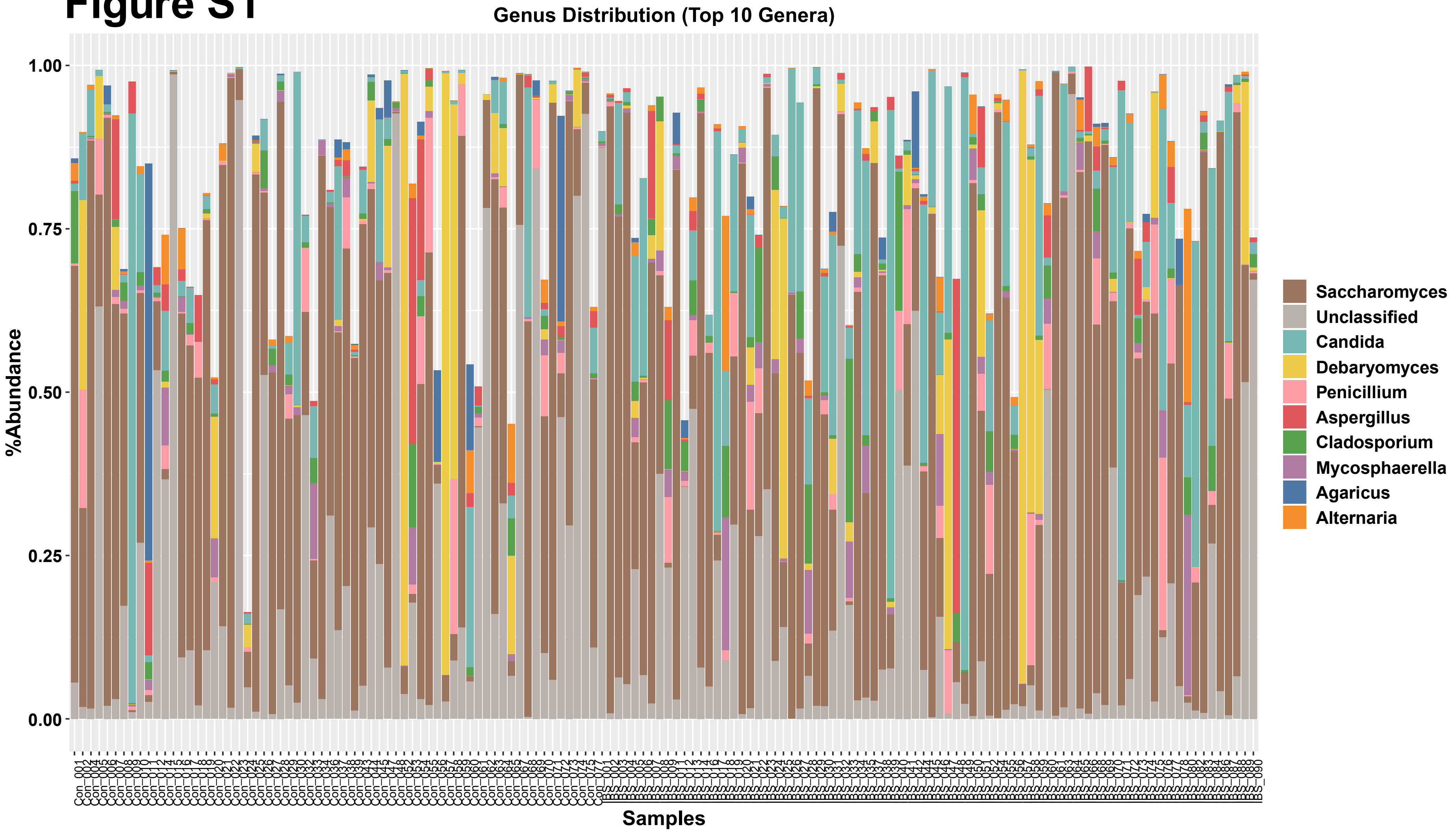

Figure S1: Distribution of top ten abundant genera across samples. Genera are arranged in decreasing order of cumulative abundance.

Figure S2

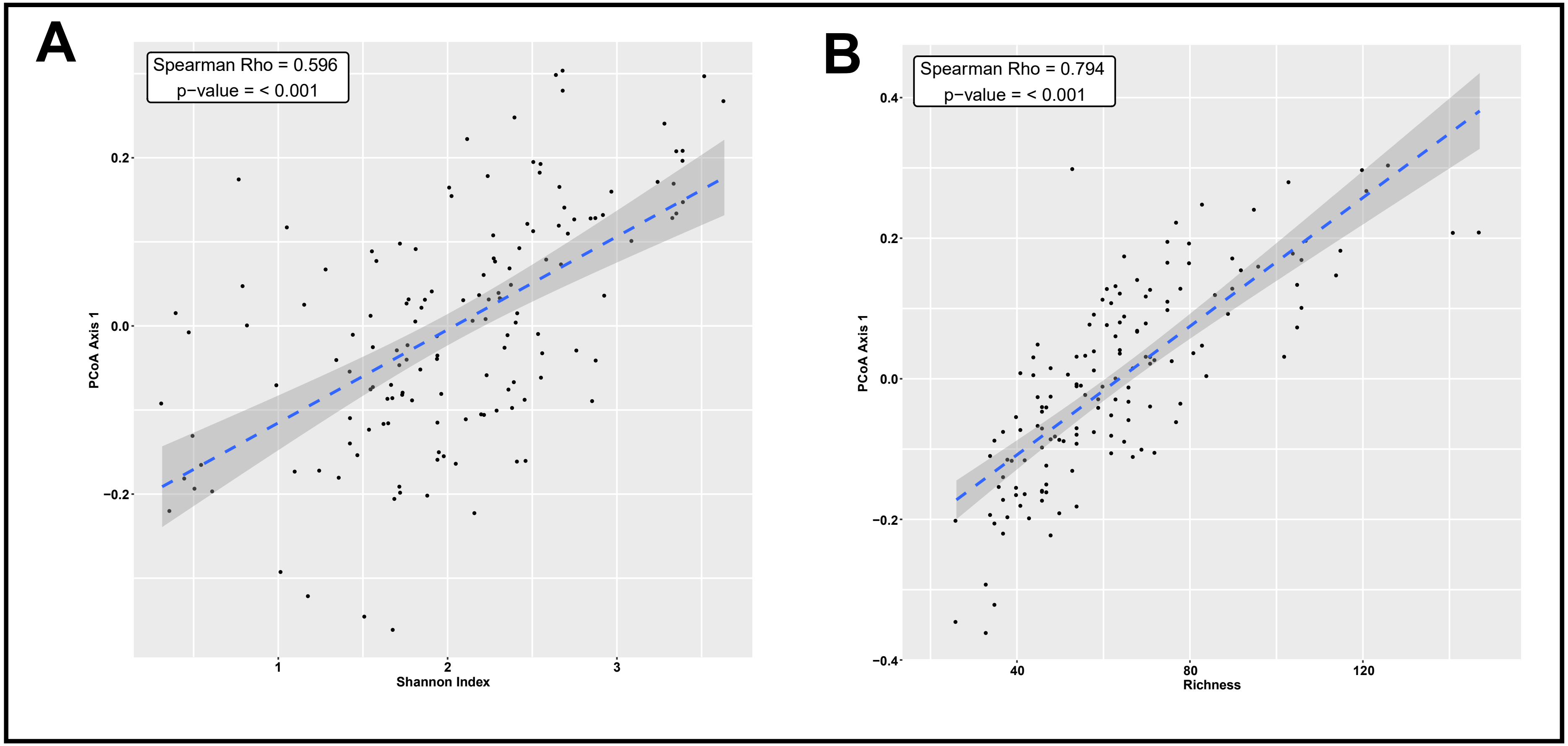

Figure S2: Association of zOTU diversity to 1st principal axis, within the analyzed population (n=144). A) Linear association between Shannon diversity of zOTUs and the first principal axis. Spearman correlation was high and significant (Rho = 0.596, p-value < 0.001). B) Linear association between Richness of zOTUs and the first principal axis. Spearman correlation was high and significant (Rho = 0.794, p-value < 0.001).

Figure S3

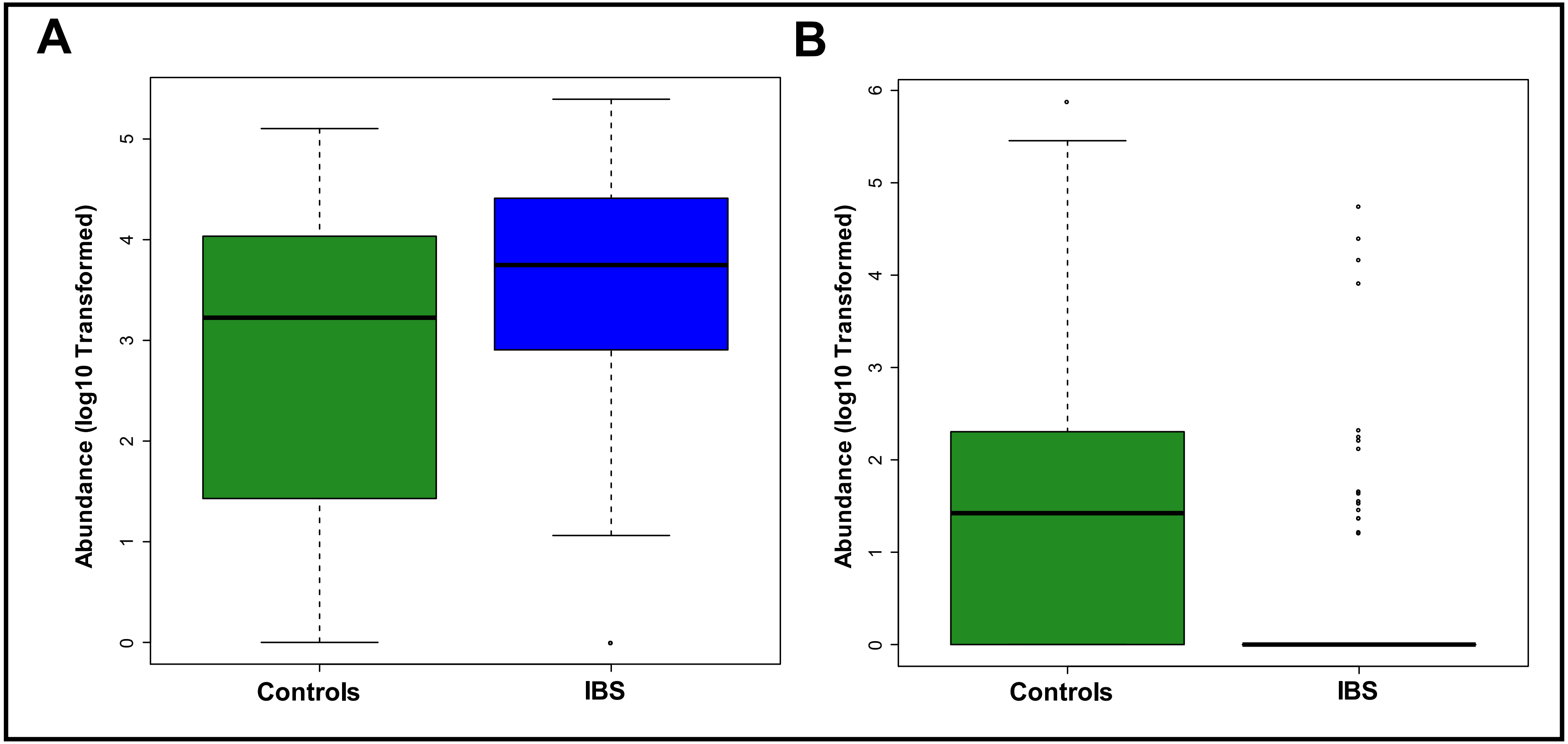

Figure S3: Boxplots showing differences between IBS and controls with the 2 significant zOTUs based on SVM models. A) Boxplot of zOTU7, which was significantly higher in IBS (Wilcoxon rank sum test, p-value < 0.05). This zOTU was classified as *Cladosporium cladosporioides*. B) Boxplot of zOTU10 was significantly lower in IBS (Wilcoxon rank sum test, p-value < 0.05). This zOTU was an unclassified fungus.

Figure S4

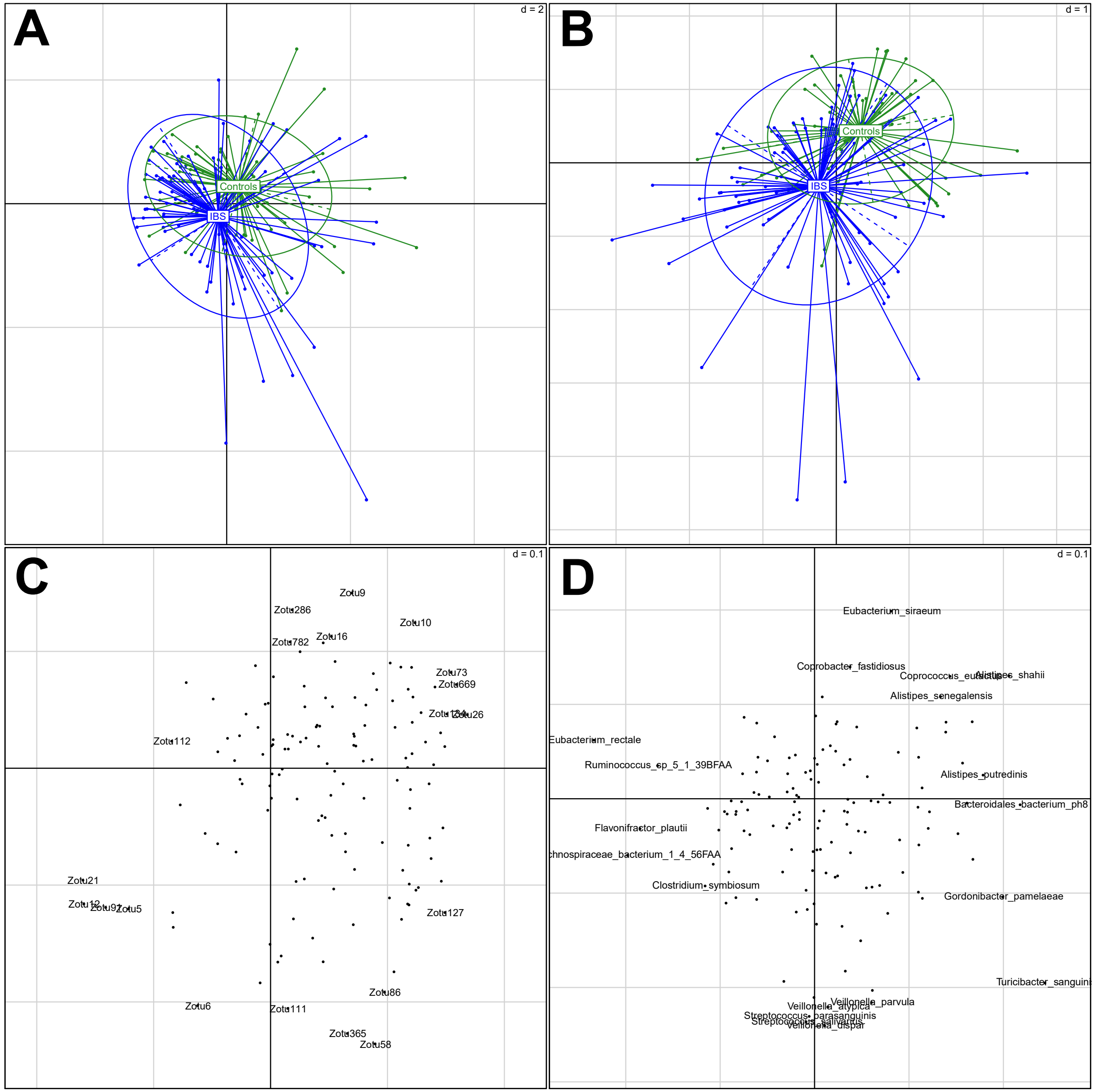

Figure S4: Co-inertia analysis plots showing co-variation between mycobiome (A) and species level metagenomic dataset (B). C: Highlights the loadings of fungal zOTUs and D highlights the loadings of bacterial species.

Figure S5

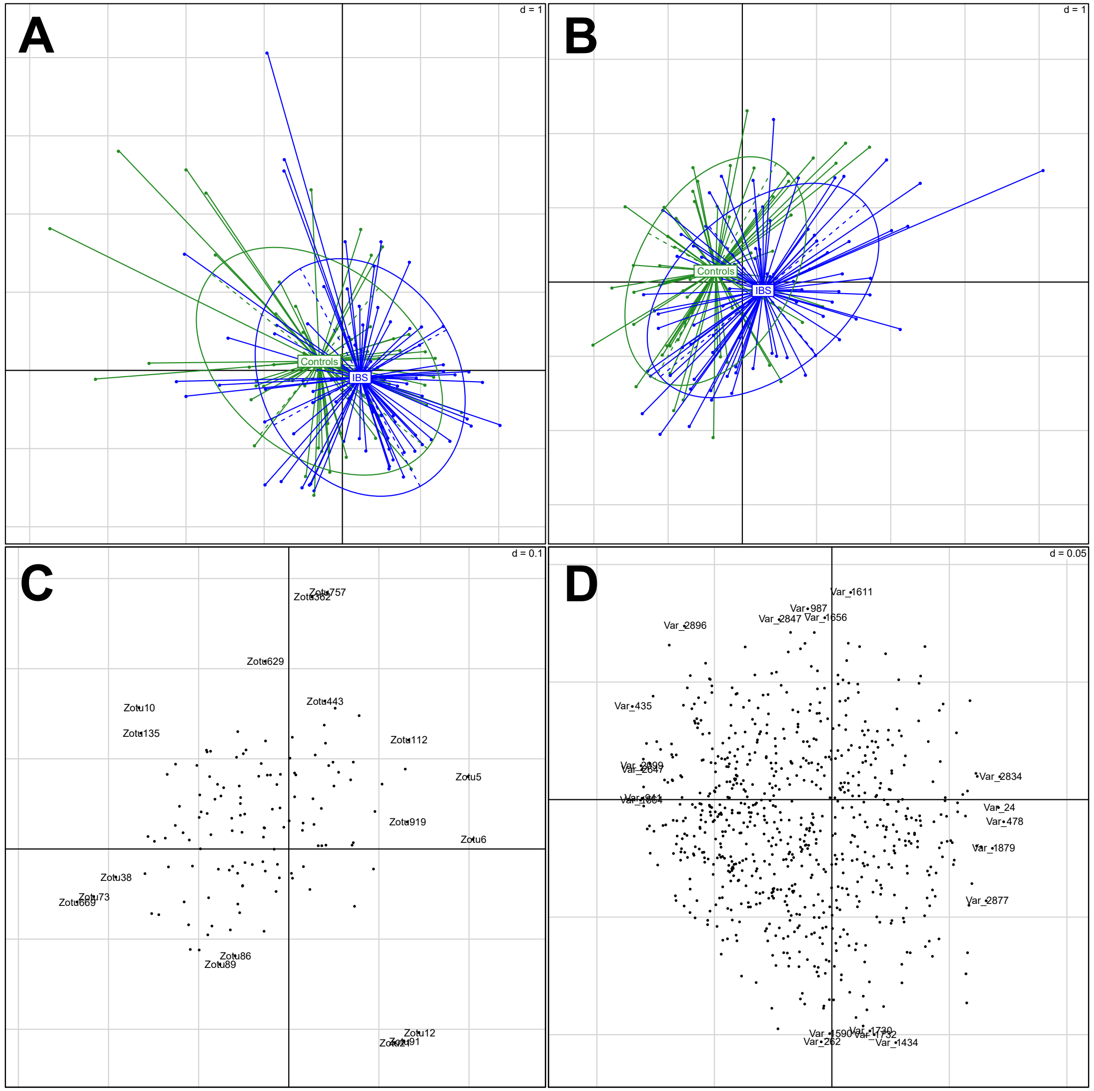

Figure S5: Co-inertia plots showing covariance between mycobiome (A) and fecal metabolome (B). C: Highlights the loadings of fungal zOTUs. D: highlights the loadings of fecal metabolites. (Metabolites corresponding to variable IDs are given in supplementary Table S3).
